# Prognostic Significance of CCDC8 in Bladder Cancer: Insights from Bioinformatics and Immunohistochemical Analysis

**DOI:** 10.7150/jca.102655

**Published:** 2025-01-01

**Authors:** Xiaojie Zhao, Xin Yu, Wenge Li, Lei Wang, Xiaodong Weng, Cheng Liu, Xiuheng Liu

**Affiliations:** 1 Department of Urology, Renmin Hospital of Wuhan University, 430060 Wuhan, Hubei, China.; 2 Department of Breast and Thyroid Surgery, Renmin Hospital of Wuhan University, 430060 Wuhan, Hubei, China.; 3 Department of Oncology, Shanghai Artemed Hospital, 200131 Shanghai, China.; 4 Department of Gynecology and Obstetrics, Renmin Hospital of Wuhan University, 430060 Wuhan, Hubei, China.

**Keywords:** pro-apoptotic coiled-coil domain containing 8 (CCDC8), bladder cancer, computational biology and bioinformatics, tumor microenvironment

## Abstract

**Background:** Pro-apoptotic coiled-coil domain containing 8 (CCDC8) has been linked to tumor progression and metastasis, yet its prognostic significance and underlying molecular mechanisms in bladder cancer remain to be elucidated.

**Materials and methods:** This study utilized raw data from public databases along with a single-center retrospective case series. We performed bioinformatics analysis and immunohistochemistry to investigate the biological landscape of CCDC8 in various tumors, with a particular focus on bladder cancer. This involved examining its expression characteristics and prognostic value. Gene function enrichment analysis was conducted to perform functional annotation, evaluate the association between bladder cancer molecular subtypes and mutation spectra, and analyze the tumor immune microenvironment to predict treatment response sensitivity.

**Results:** Our study identified CCDC8 as a novel prognostic marker for bladder cancer. We observed that high CCDC8 expression correlates with poor prognosis and a suboptimal response to immunotherapy in bladder cancer. CCDC8 was implicated in regulating tumor immune status, metabolic activity, and cell cycle-related signaling pathways, thereby influencing the biological behavior of tumor cells. Additionally, CCDC8 contributed to the suppression of the immune microenvironment, diminishing anti-tumor immune responses. Comprehensive characterization of CCDC8 was applied to prognostic prediction in bladder cancer, indicating that targeting CCDC8 may be a potential therapeutic strategy.

**Conclusions:** These findings suggest that CCDC8 serves as an independent biomarker for predicting prognosis and immunotherapy efficacy for bladder cancer. Further investigation into its specific molecular mechanisms may offer new therapeutic strategies for treating bladder cancer.

## Introduction

A significant public health problem, bladder cancer (BLCA) is the second most common cancer of the urinary system, and its incidence is increasing worldwide^1^, ^2^. Despite advancements in surgical techniques, systemic therapies, and innovative treatments such as immunotherapy, overall survival rates for BLCA remain suboptimal^3^. A major challenge in BLCA treatment is the development of resistance to immunotherapy, which limits the efficacy of these promising interventions^4^. Therefore, it is essential to deepen our understanding of the mechanisms in molecular biology driving BLCA and identify novel therapeutic targets and biomarkers that can enhance patient outcomes and enable more effective treatment strategies.

The coiled-coil domain-containing protein family (CCDC) encompasses a range of proteins integral to various cellular processes, including cell cycle regulation, signaling pathways, and transcription^5^. Initially, CCDC8, along with CUL7 and OBSL1, was suggested to be one of the causative genes of Three M syndrome^6^. Increasing research has highlighted the relationship between CCDC8 and tumorigenesis, demonstrating its role as a tumor suppressor in various cancers, including breast^7^ , renal^8^ , and lung cancers^9^. On the other hand, a recent study has confirmed CCDC8 is correlated with tumor chemotherapy resistance^10^.Nevertheless, the specific role and mechanism of CCDC8 in bladder cancer remain unexplored. Given its crucial role in other malignancies, investigating potential implications of CCDC8 in bladder cancer is imperative.

Our study seeks to elucidate the prognostic significance of CCDC8 in bladder cancer by examining its expression patterns, immunological effects in the tumor immune environment, and molecular landscape. Through constructing and validating predictive models, we aim to offer novel insights that could pave the way for new therapeutic strategies in bladder cancer.

## Materials and methods

### Patients and specimens

Cohort1: Gene expression data (RNA-FPKM), gene mutation information, and clinicopathological data were obtained for 408 TCGA-BLCA samples. The gene IDs were converted to gene symbols^11^. When multiple probes matched a single gene symbol, the average value was used to represent the expression level. We included all patients from the TCGA-BLCA cohort with available transcriptomic data and clinical information, as the original datasets lacked exclusion criteria for comorbidities or other diseases. We classified cases into high and low CCDC8 expression groups based on the median mRNA expression within the cohort. Detailed patient characteristics in cohort 1 are provided in Table S1.

Cohort2: A total of 100 sections for immunohistochemistry from 100 different bladder cancer patients were collected at Renmin Hospital of Wuhan University between February 2018 and October 2022. This study received ethical approval from the Institutional Ethics Committee of Renmin Hospital of Wuhan University (WDRY2022-K077). Clinical features, including age, sex, and clinical stages based on the 8th Edition of the AJCC Staging System for bladder cancer, were recorded. The inclusion criteria were patients with pathologically confirmed bladder cancer and complete clinical data, while exclusion criteria included other malignancies, severe systemic diseases, poor tissue quality, or incomplete data. The characteristics of patients in cohort 2 are shown in Table S2.

Cohort3: The BLCA immunotherapy-related cohort, using the same classification criteria as Cohort 1, included 348 BLCA patients treated with atezolizumab, with detailed clinical information and comprehensive expression data obtained via the R package for IMvigor210 CoreBiologies^12^. The characteristics of patients in cohort 3 are provided in Table S3.

### Survival analyses

We conducted survival analyses using the Kaplan-Meier method with the survival R package. To evaluate the association between the prognosis of patients with bladder cancer (BLCA) and basic clinical information such as age, molecular subtype, stage of tumor, and CCDC8 expression, a univariate Cox regression analysis was employed. Clinical factors and CCDC8 expression levels were found to be significant prognostic indicators (p < 0.05) in the univariate. Subsequently, we used a multivariable Cox regression model and the survival R package to conduct Cox regression analysis.

### Screening of differentially expressed genes and functional analysis

We used the R package limma (v3.54.0) to identify DEGs and the criteria for selection were set at p < 0.05 and fold change (FC) > 1.5 for both upregulated and downregulated DEGs. Gene Ontology enrichment (GO), KEGG pathway enrichment, and hallmark gene set enrichment analyses were conducted through the ClusterProfiler package.

### Annotation for molecular subtype of bladder cancer

We employed the ConsensusMIBC R package to classify the molecular subtypes of bladder cancer across various classification systems, including CIT, MDA, Lund, Baylor, TCGA, and UNC subtypes.

### Immune analysis of cell infiltration

To assess the infiltration of immune cells within tumor samples, we employed the CIBERSORT algorithm using the cibersort R package. This method allowed us to quantify the existence and percentage of 22 specific kinds of immune cells within the tumor microenvironment. By leveraging this algorithm, we gained detailed information of tumor immune cell composition, facilitating a deeper insight into the immune landscape and its potential impact on tumor behavior and patient prognosis.

### Prediction of chemotherapy response

To estimate the IC50 values of numerous chemotherapeutic drugs, we used the "pRRophetic" R package, which predicts drug sensitivity based on gene expression profiles. Additionally, the DrugBank database (https://go.drugbank.com/) was utilized to screen for drug-target genes. This approach allowed us to link predicted drug responses with specific genetic alterations, aiding in the identification of potential therapeutic targets.

### Immunohistochemical analysis

As described in our previous study^13^, IHC staining was conducted by two independent pathologists. The staining results were evaluated based on the percentage and intensity of positively stained tumor cells. The intensity of CCDC8 expression was scored as follows: 0 (negative), 1 (weak positive, light brown), 2 (moderate positive, brown), and 3 (strong positive, dark brown). We used the following formula for protein staining score = percentage score × intensity score. The cut-off value for the protein staining score of CCDC8 was established by calculating the cut-off values between all possible upper and lower quartiles and selecting the optimal threshold. The antibody used in this assay was anti-CCDC8 (Cat No. 27194-1-AP, Proteintech, China).

### Statistical analysis

We used Pearson correlation analysis to investigate the relationships between variables. For comparing continuous variables between binary groups with a normal distribution, we conducted a t-test. Additionally, when comparing more than two groups, we applied the Kruskal-Wallis test to identify differences. To determine statistically significant differences, we used the log-rank test, while the Kaplan-Meier method was employed to generate survival curves for subgroups within each dataset. All statistical analyses were performed using SPSS 22.0, SangerBox (http://sangerbox.com), and R studio v4.0.0. Finally, we calculated P values as two-sided, considering values less than 0.05 to be statistically significant.

## Results

### Expression, prognostic value, and mutation characteristics of CCDC8 in pan-cancer

**Figure 1:**
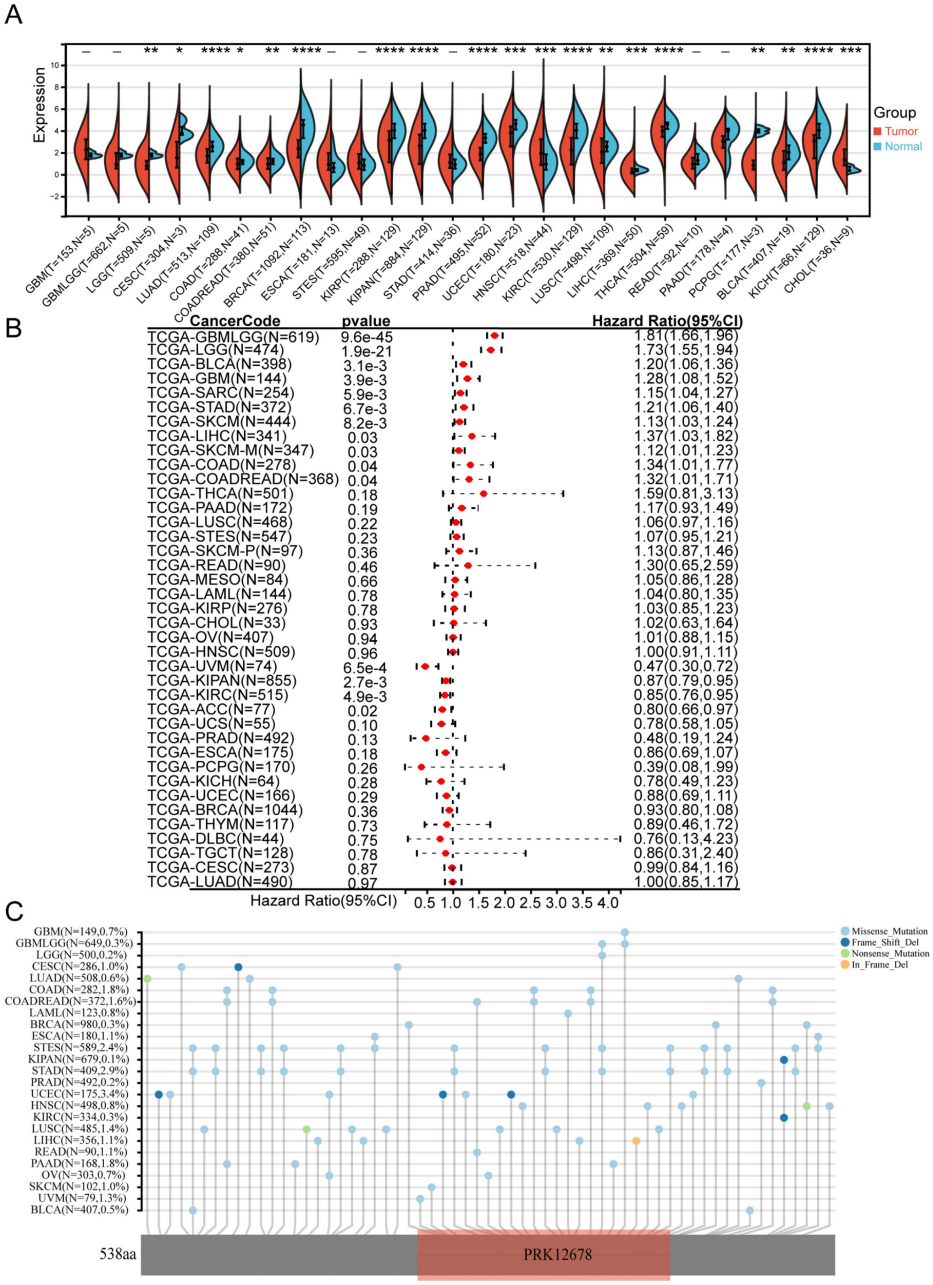
** Expression, prognostic value, and mutation characteristics of CCDC8 in Pan-cancer.** (A) CCDC8 expression across 34 cancer types compared to normal tissues, as analyzed using TCGA data. (B) Forest plot illustrating the associations between CCDC8 expression and overall survival in various cancer types (pan-cancer). (C) Map position of CCDC8 mutations in pan-cancer, highlighting mutation frequency and locations within the gene. *P < 0.05, **P < 0.01, ***P < 0.001, and ****P < 0.0001.

We initially examined the expression levels of CCDC8 in normal and tumor samples across 34 different cancer types using data from TCGA. Our findings indicated that CCDC8 was significantly downregulated in 17 tumor types, including bladder cancer (BLCA), and upregulated in only 2 cancer types, specifically head and neck squamous cell carcinoma (HNSC) and cholangiocarcinoma (CHOL) (Figure 1A). Subsequently, we assessed the prognostic value of CCDC8 in TCGA pan-cancer datasets using univariate Cox regression analysis. Notably, the results for bladder cancer (BLCA) demonstrated significant statistical relevance, indicating that high CCDC8 expression levels may serve as an independent predictor of poor prognosis in BLCA patients (Figure 1B). Moreover, we investigated the mutation characteristics of CCDC8 across various cancer types, focusing on mutation frequency and types. There were significant differences in the mutation frequency and types of CCDC8 among different cancers. For instance, uterine corpus endometrial carcinoma (UCEC) exhibited the highest mutation frequency of CCDC8, whereas other cancer types showed relatively lower frequencies. In bladder cancer (BLCA), the mutation frequency of CCDC8 was 0.5%, with missense mutations being the predominant type. Although the mutation frequency is low, these mutations may significantly impact the development and progression of bladder cancer (Figure 1C).

### Prognostic value of CCDC8 expression in bladder cancer

**Figure 2:**
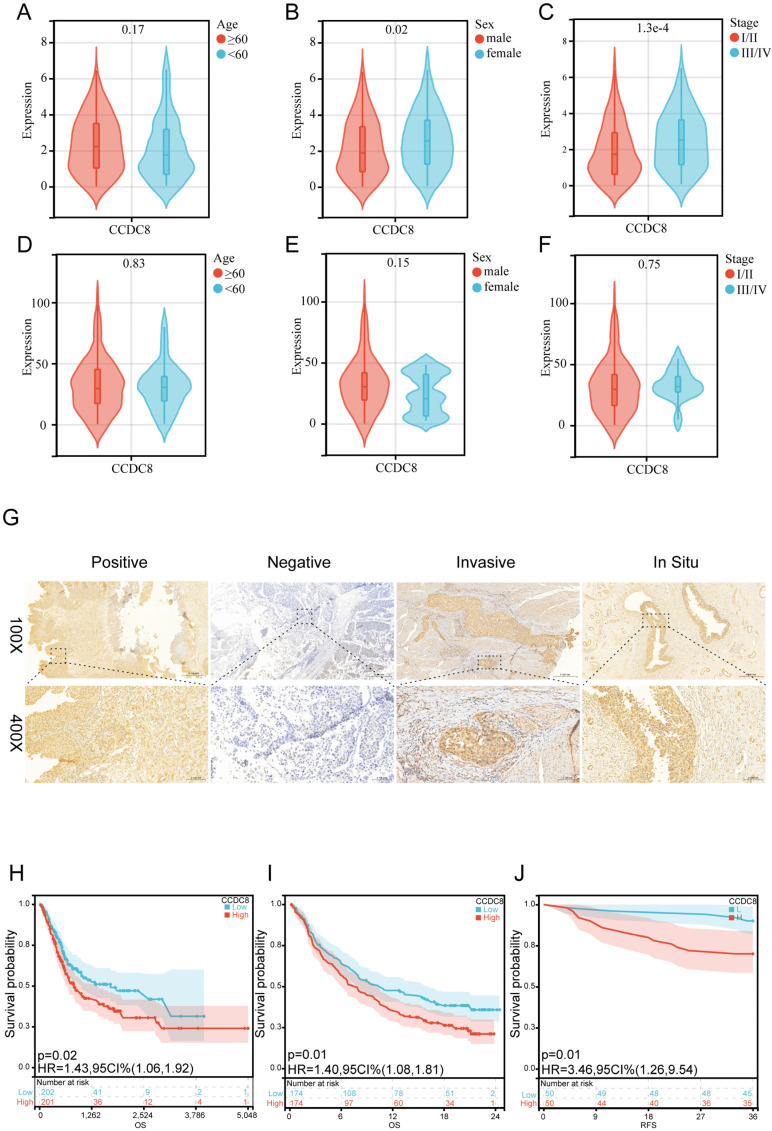
** Prognostic value of the CCDC8 expression in Bladder Cancer.** (A-C) CCDC8 expression in different (A) Age, (B) Sex and (C) Stage in cohort1. (D-F) CCDC8 expression in different (D) Age, (E) Sex and (F) Stage in cohort2. (G) Representative immunohistochemistry images for CCDC8 in bladder cancer (100X, bar=500uM and 400X,bar=100uM). (H-J) OS for CCDC8 in (H) cohort1 and (I) cohort3, RFS for CCDC8 in (J) cohort2.

Firstly, we analyzed the expression and clinical features of CCDC8 in patients from cohort 1. The results indicate that CCDC8 expression is significantly higher in females and in advanced cancer stages (III/IV) (Figure 2A-C). In contrast, cohort 2 shows no significant differences in CCDC8 expression based on age, sex, or cancer stage (Figure 2D-F). These findings suggest potential differences in CCDC8 expression patterns between the two cohorts, warranting further investigation. To further verify the association of CCDC8 expression level with the prognosis of BLCA patients in cohort 2, we evaluated the expression of CCDC8 in 100 BLCA patients using IHC (Figure 2G). The IHC results showed that CCDC8 was diffusely localized in the cytoplasm and nuclear of BLCA cells. In summary, the Kaplan-Meier survival curves indicate that high CCDC8 expression is associated with poorer survival outcomes, including both overall survival and recurrence-free survival, across the three cohorts (Figure 2H-J). These findings underscore the potential prognostic significance of CCDC8 expression in bladder cancer.

### Functional annotation of differentially expressed genes

**Figure 3:**
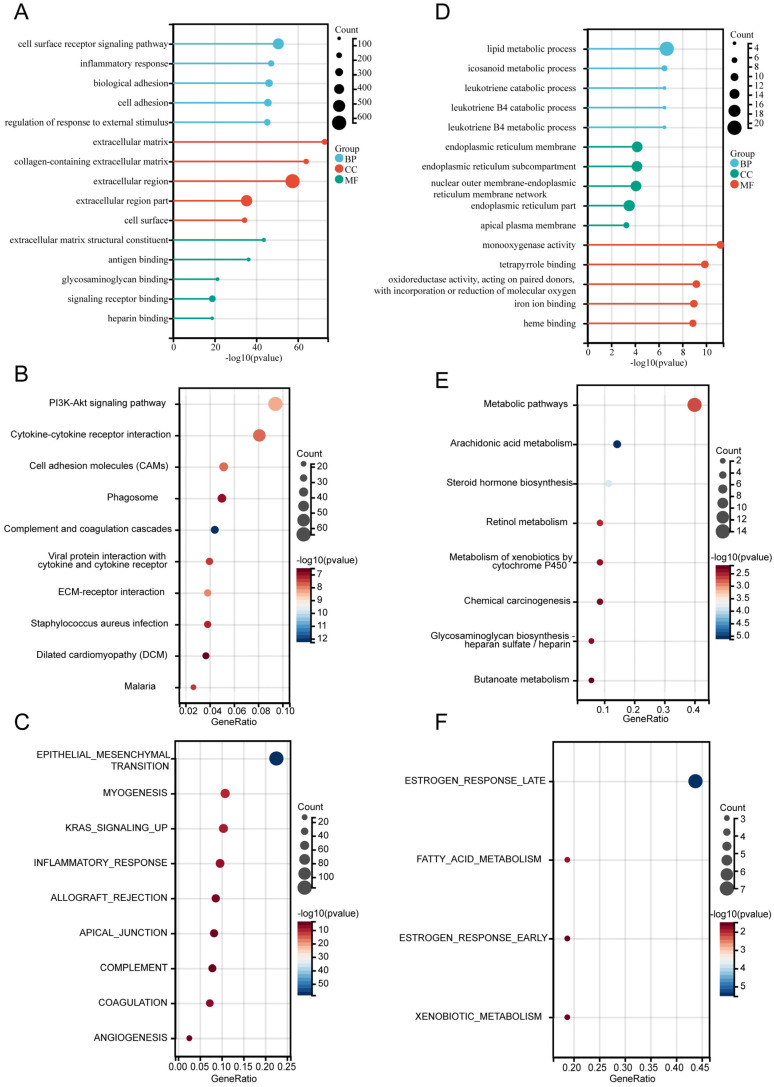
** Functional annotation of differentially expressed genes.** (A-C) Functional enrichment analysis of upregulated DEGs: (A) Gene Ontology (GO) terms, (B) KEGG pathways, and (C) Hallmark gene sets. (D-F) Functional enrichment analysis of downregulated DEGs: (D) Gene Ontology (GO) terms, (E) KEGG pathways, and (F) Hallmark gene sets.

For the differentially expressed genes (DEGs) in cohort 1, the Limma package was used for analysis, and the results were visualized through a heat map and volcano plot, illustrating the expression of differential genes (Figure S1A-B). Subsequently, functional annotations of DEGs were performed. GO analysis revealed that upregulated DEGs were significantly enriched in the “Cell surface receptor signaling pathway”(BP), “Extracellular matrix”(CC), and “Extracellular matrix structural constituent”(MF) (Figure 3A). KEGG pathway analysis indicated that the top enriched pathways included the PI3K-Akt signaling pathway, cytokine-cytokine receptor interaction, cell adhesion molecules (CAMs), and phagosomes. Other notable pathways were complement and coagulation cascades, ECM-receptor interaction, and Staphylococcus aureus infection (Figure 3B). The hallmark gene sets enriched among upregulated DEGs included epithelial-mesenchymal transition (EMT), allograft rejection, myogenesis, KRAS signaling, inflammatory response, apical junction, complement, coagulation, and angiogenesis (Figure 3C).

For the downregulated DEGs, GO analysis suggested that “Lipid metabolic process” in BP, “Endoplasmic reticulum membrane” in CC, and “Monooxygenase activity” in MF (Figure 3D) were the most substantially enriched pathways. The top enriched KEGG pathways were metabolic pathways, arachidonic acid metabolism, steroid hormone biosynthesis, and retinol metabolism. Additional pathways included the metabolism of xenobiotics by cytochrome P450, chemical carcinogenesis, and glycosaminoglycan biosynthesis-heparan sulfate/heparin (Figure 3E). The hallmark gene sets enriched among downregulated DEGs included estrogen response (late and early), fatty acid metabolism, and xenobiotic metabolism (Figure 3F).

These findings offer insights into the functional roles and pathways influenced by the differential expression of genes in bladder cancer.

### Association of CCDC8 with molecular subtypes and differentiation pathways in bladder cancer

**Figure 4:**
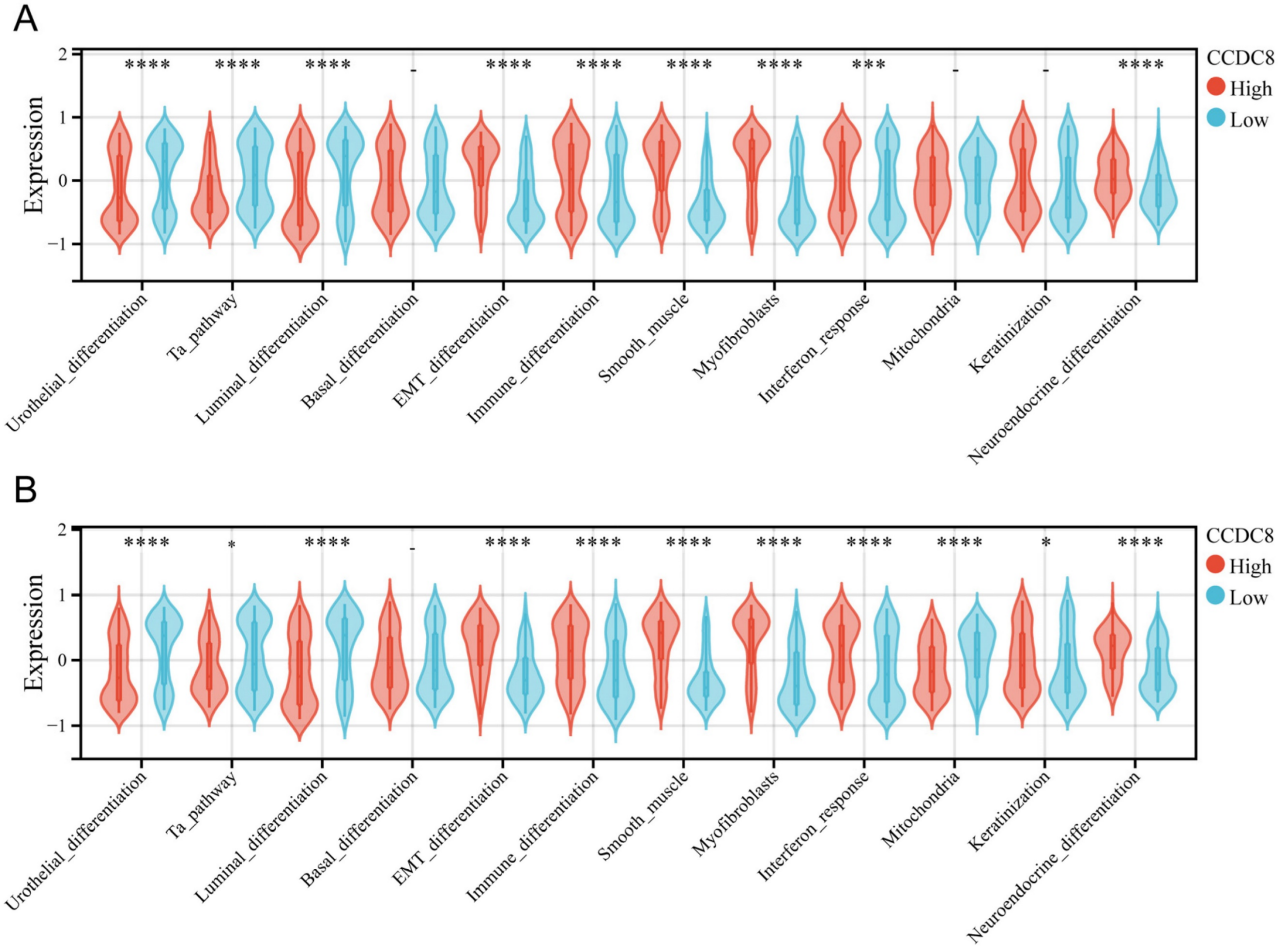
** Association of CCDC8 with molecular subtypes and differentiation pathways in Bladder Cancer.** Differential expression of bladder cancer-related signatures between high and low CCDC8 expression groups in (A) cohort 1 and (B) cohort 3. *P < 0.05, ***P < 0.001, and ****P < 0.0001.

The molecular subtypes of bladder cancer are crucial for determining prognosis and response to chemotherapeutic agents, immunotherapeutic strategies, and other therapeutic interventions. In cohort 1, we analyzed the correlation between CCDC8 expression level and the bladder cancer molecular subtypes. These results revealed that all six molecular subtype identification algorithms consistently indicated that the high CCDC8 group has a higher incidence of basal type bladder cancer (Table S4). Additional examination of molecular features indicated that the high CCDC8 group exhibited higher levels of EMT differentiation, immune differentiation, smooth muscle, interferon response, and neuroendocrine differentiation. Conversely, these patients showed lower levels of urothelial differentiation, Ta pathway, and luminal differentiation (Figure 4A). A similar analysis was conducted in cohort 3 (Table S5), confirming these findings. In this cohort, the high CCDC8 expression group also displayed increased immune differentiation, EMT differentiation, myofibroblasts, smooth muscle, interferon response, and neuroendocrine differentiation, along with reduced urothelial differentiation, luminal differentiation, and mitochondrial activity (Figure 4B).

These results highlight the significant role of CCDC8 as a crucial regulator in the differentiation and growth of bladder cancer. The strong link between high CCDC8 expression and basal type bladder cancer, as well as various differentiation pathways, highlights its potential as both a biomarker and a therapeutic target. Further investigation into the mechanistic roles of CCDC8 could provide valuable insights for developing targeted therapies and improving prognostic results for bladder cancer patients.

### The relationship between CCDC8 expression and the tumor immune microenvironment

**Figure 5:**
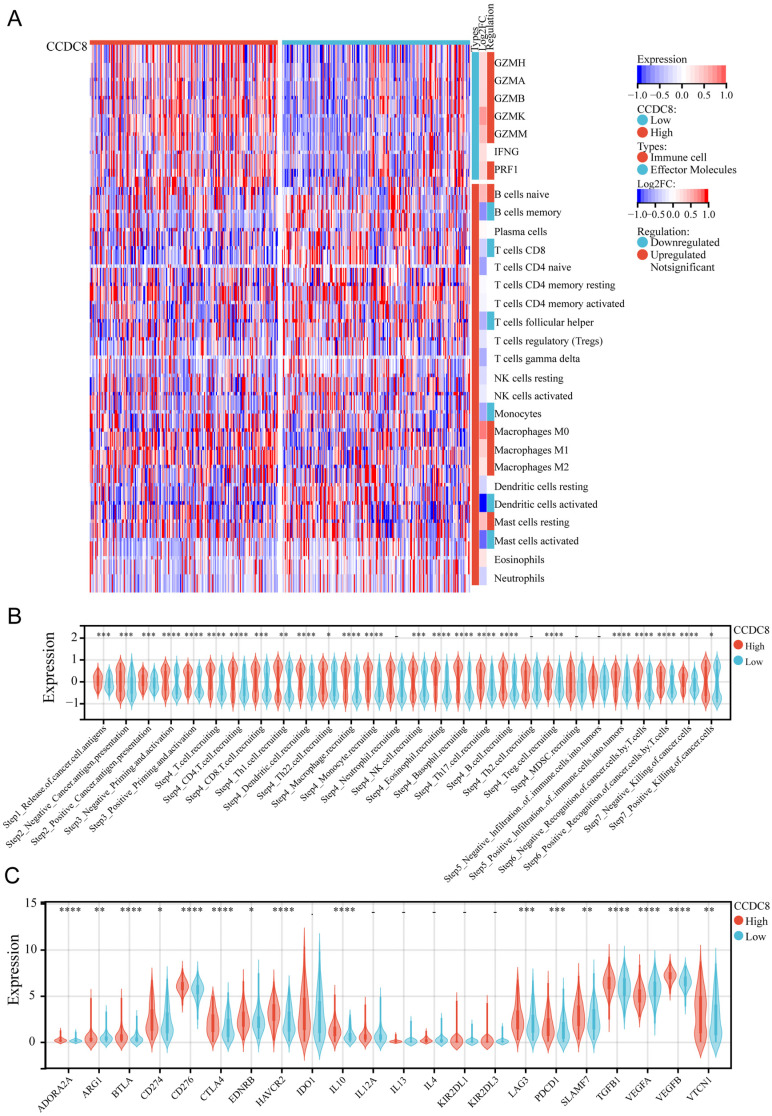
** The relationship between CCDC8 expression and the tumor immune microenvironment in cohort1.** (A) Heatmap showing the distribution of immune cells between high and low CCDC8 expression groups. (B-C) Correlation violin plots depicting the differences between high and low CCDC8 expression groups in (B) the immune cycle and (C) immune checkpoints. *P < 0.05, **P < 0.01, ***P < 0.001, and ****P < 0.0001.

We evaluated the expression levels and infiltration status of tumor-infiltrating immune cells, the activity of tumor immunity cycle, and immune checkpoints in cohort 1 to investigate the potential link between CCDC8 and immunological features. First, we deconvolute the infiltration of immune cells in the tumor immune microenvironment (TIME) using the CIBERSORT algorithm. For effector molecules, patients with high CCDC8 expression exhibited higher levels of GZMH, GZMA, GZMB, GZMK, GZMM, and PRF1 compared to those with low CCDC8 expression, while IFNG levels did not show significant changes. Regarding immune cell infiltration, lower infiltration of memory B cells, CD8+ T cells, follicular helper T cells, monocytes, activated dendritic cells (DCs), and activated mast cells was observed in patients with high CCDC8 expression. Conversely, higher infiltration levels of naive B cells, M0 macrophages, M1 macrophages, M2 macrophages, and resting mast cells were observed in these patients (Figure 5A). The high CCDC8 group could activate most steps in the immunity cycle, except for neutrophil recruiting (Step 4), Th2 cell recruiting (Step 4), MDSC recruiting (Step 4), and negative infiltration of immune cells into tumors (Step 5) (Figure 5B).

In cohort1, CCDC8 expression was positively correlated with the expression of various immune checkpoints, including inhibitory checkpoints (BTLA, CTLA4, LAG3, PDCD1), stimulatory checkpoints (CD276, CD274), and immune modulators (IDO1, IL10, TGFB1, ARG1, EDNRB, HAVCR2). However, CCDC8 was negatively correlated only with VEGFA expression. This suggests that CCDC8 may play a significant role in regulating tumor immune evasion by modulating these immune checkpoint genes. (Figure 5C).

**Figure 6:**
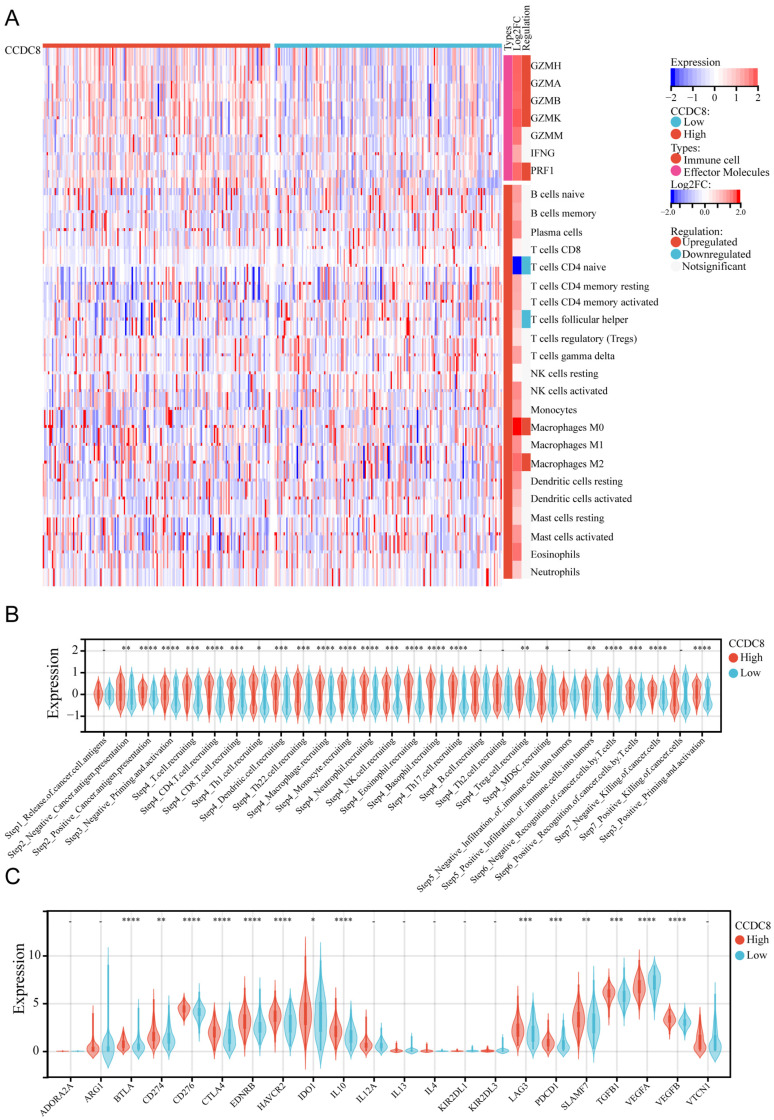
** The relationship between CCDC8 expression and the tumor immune microenvironment in cohort 3.** Heatmap showing the distribution of immune cells between high and low CCDC8 expression groups. (B-C) Correlation violin plots depicting the differences between high and low CCDC8 expression groups in (B) the immune cycle and (C) immune checkpoints. *P < 0.05, **P < 0.01, ***P < 0.001, and ****P < 0.0001.

The same analysis was conducted for cohort 3. We observed that the expression of effector molecules, such as GZMH, GZMA, GZMB, GZMK, and PRF1, was significantly enriched, except for GZMM and IFNG. In terms of immune cell infiltration, there was less infiltration of naive CD4+ T cells and follicular helper T cells, and more infiltration of M0 macrophages and M2 macrophages (Figure 6A). Similarly, the high CCDC8 group was able to activate most steps in the immunity cycle, except for the release of cancer cell antigens (Step 1), B cell recruiting (Step 4), Th2 cell recruiting (Step 4) negative infiltration of immune cells into tumors (Step 5), and positive killing of cancer cells (Step 7) (Figure 6B). In cohort3, similar to cohort1, CCDC8 expression was positively correlated with various immune checkpoints and negatively correlated with VEGFA expression (Figure 6C).

In summary, our findings indicate that CCDC8 is intricately involved in modulating the tumor immune microenvironment.

### Exploring the function of CCDC8 in TIME of pan-cancer

Since CCDC8 was found to be associated with various immune effectors and specific patterns of immune cell infiltration, as well as activating multiple steps in the immunity cycle, we aimed to explore the association between CCDC8 expression patterns and the tumor immune microenvironment in other cancers. First, we used a heatmap to display the expression correlations of various immune-related genes (including chemokines, receptors, MHC molecules, immunoinhibitors, and immunostimulators) across multiple cancer types. The strength of these correlations varied significantly between different tumors, showing notable heterogeneity. Specifically, bladder cancer (BLCA) demonstrated a significant positive correlation with numerous immune-related genes, revealing its important role in regulating the tumor immune microenvironment (Figure S2A). Next, the relationship between CCDC8 and immune checkpoint genes in a pan-cancer context was analyzed. The heatmap revealed significant heterogeneity in the immune-related genes expression within different cancer types, underscoring the complexity of immune regulation within the tumor microenvironment. Notably, cancers such as prostate adenocarcinoma (PRAD), pancreatic adenocarcinoma (PAAD), rectum adenocarcinoma (READ), and bladder cancer (BLCA) showed that these genes are crucial in regulating the tumor immune microenvironment, suggesting specific functions and regulatory mechanisms in different cancer types (Figure S2B).Lastly, the heatmap highlighted significant heterogeneity in the infiltration of immune cells and their association with tumor characteristics across different cancer types. This further emphasizes the complexity of immune regulation in the tumor immune microenvironment. Once again, cancers like PRAD, PAAD, READ, and BLCA demonstrated that these immune cells serve a vital function in regulating the tumor immune microenvironment, suggesting that distinct functions and regulatory mechanisms may be attributed to these cells in various cancer types. (Figure S2C).

### Predictive role of CCDC8 in immunotherapy for bladder cancer

**Figure 7:**
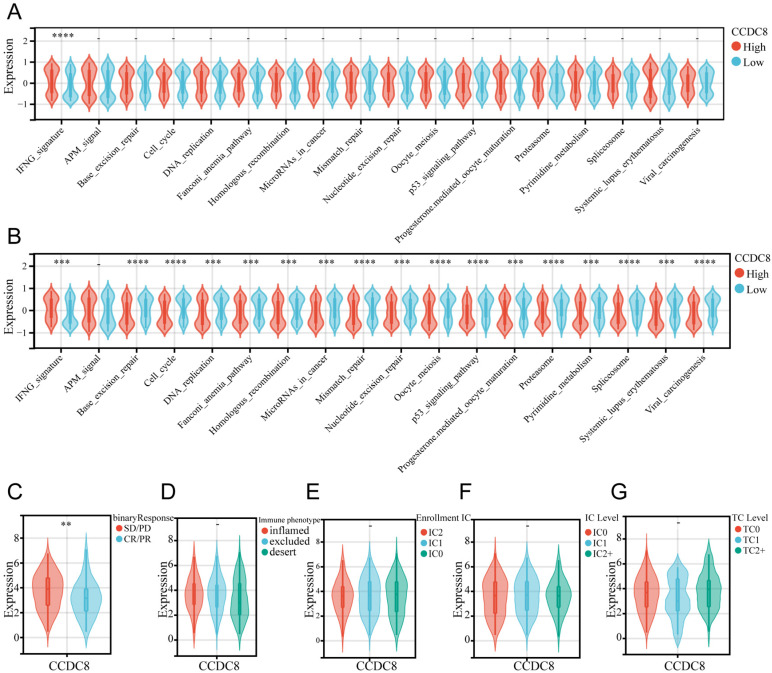
** CCDC8 predicts immunotherapy response.** (A) relationship between CCDC8 and immune response-related pathways in cohort1 and(B) cohort3. (C-G) CCDC8 expression in patients with (C) different clinical response of tumor immunotherapy, (D) immune cell infiltration types, (E) enrollment IC, (F) IC levels and (G) TC levels. **P < 0.01, ***P < 0.001, and ****P < 0.0001.

To further explore the predictive role of CCDC8 in immunotherapy, we analyzed the performance of immunotherapy-related pathways in cohort1 and cohort3. In cohort1, only the IFNG signaling pathway was significantly activated (Figure 7A). However, in cohort3, CCDC8 similarly significantly activated the IFNG signaling pathway but inhibited multiple immune-related pathways, including base excision repair, cell cycle, DNA replication and so on (Figure 7B). This differential regulation suggests that tumors with high CCDC8 expression might exhibit lower sensitivity to immunotherapy and potential therapeutic resistance. Moreover, data from cohort 3 showed that high CCDC8 expression is significantly associated with poorer treatment response (SD/PD) (Figure 7C) but is not significantly related to tumor immune phenotypes, immune cell infiltration classifications, IC levels, or TC levels (Figure 7D-G). Overall, the identification of CCDC8 as a predictive marker for immunotherapy highlights its importance in enhancing personalized treatment approaches in cancer therapy.

### The role of CCDC8 in radiotherapy, chemotherapy, and targeted therapy for bladder cancer

**Figure 8:**
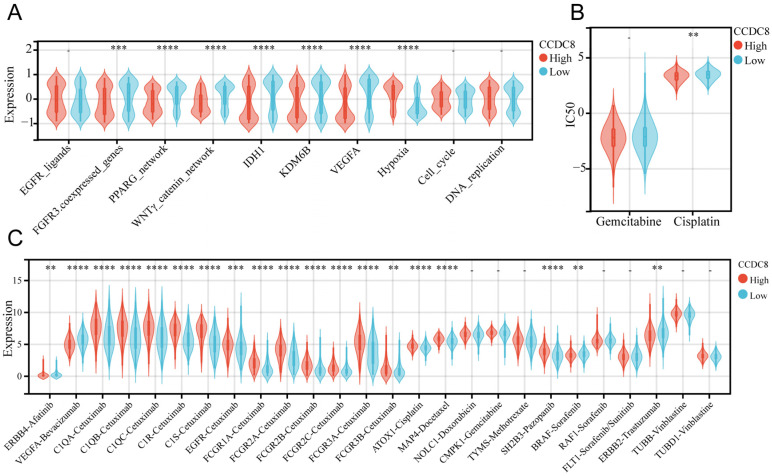
** The relationship between CCDC8 expression and the signature of radiotherapy, chemotherapy, and targeted therapy in cohort 1.** (A-C) Differential expression of therapeutic signatures between high and low CCDC8 groups: (A) targeted therapy and radiotherapy, (B) IC50 values for gemcitabine and cisplatin therapy, (C) drug-target genes. **P < 0.01, ***P < 0.001, and ****P < 0.0001.

**Figure 9:**
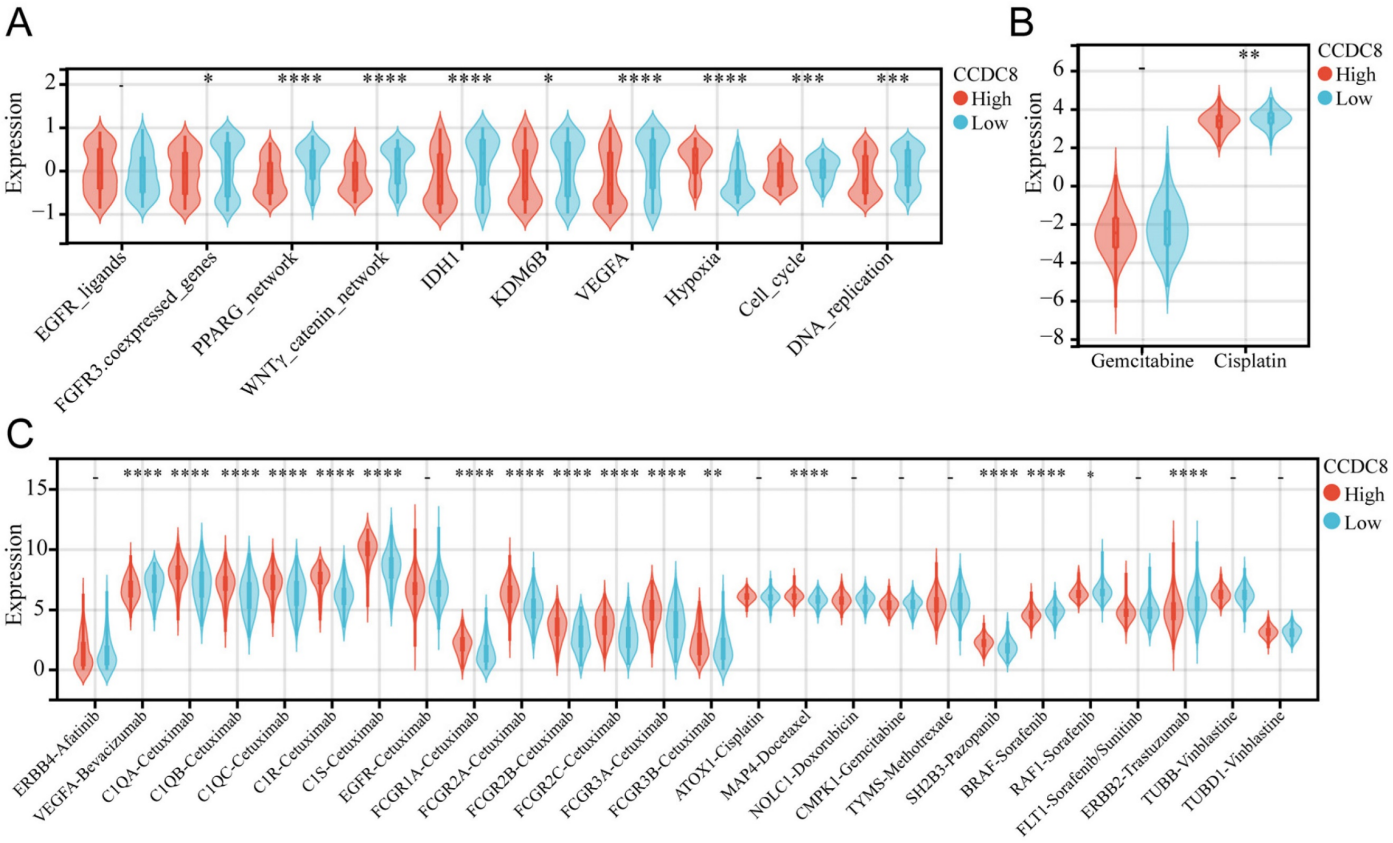
** The relationship between CCDC8 expression and the signature of radiotherapy, chemotherapy, and targeted therapy in cohort 3.** (A-C) Differential expression of therapeutic signatures between high and low CCDC8 groups: (A) targeted therapy and radiotherapy, (B) IC50 values for gemcitabine and cisplatin therapy, (C) drug-target genes. *P < 0.05, **P < 0.01, ***P < 0.001, and ****P < 0.0001.

We analyzed the co-expression of CCDC8 with several radiotherapy-related pathways and target genes. In cohort1, CCDC8 showed a significant negative correlation with target genes such as FGFR3, IDH1, KDM6B, and VEGFA, as well as with pathways including the PPARG network and WNT/β-catenin network. Conversely, CCDC8 was positively correlated with the hypoxia pathway (Figure 8A). Applying the same analysis to cohort3, we found that, in addition to the previous results, CCDC8 was also significantly negatively correlated with the cell cycle and DNA replication pathways (Figure 9A). Using pRRophetic algorithm, we calculated IC50 values and found that the high CCDC8 group in both cohort 1 and cohort 3 exhibited greater sensitivity to cisplatin, a chemotherapeutic agent frequently used in the therapy of bladder cancer. On the other hand, there was no significant difference in sensitivity to gemcitabine between the high and low CCDC8 groups (Figure 8B, Figure 9B). Additionally, results obtained from the DrugBank database revealed distinct patterns of drug sensitivity based on CCDC8 expression levels. In the high CCDC8 group, target genes associated with EGFR-targeted drugs and various chemotherapy agents were significantly elevated, suggesting greater sensitivity to these treatments. On the other hand, in the low CCDC8 expression group, target genes related to anti-angiogenesis drugs and HER2-targeted therapies were significantly higher, indicating increased sensitivity to these drugs. Interestingly, while both Pazopanib and Sorafenib target the BRAF/RAF pathways, our results demonstrated opposite patterns of sensitivity for these two drugs (Figure 8C). To further validate these findings, we conducted the same analysis in cohort 3, which exhibited similar results (Figure 9C). Future research should focus on validating these results and exploring the mechanistic role of CCDC8 in modulating treatment responses, aiming to develop more personalized and effective therapeutic strategies for bladder cancer.

### Mutational landscape in bladder cancer stratified by CCDC8 expression levels

**Figure 10:**
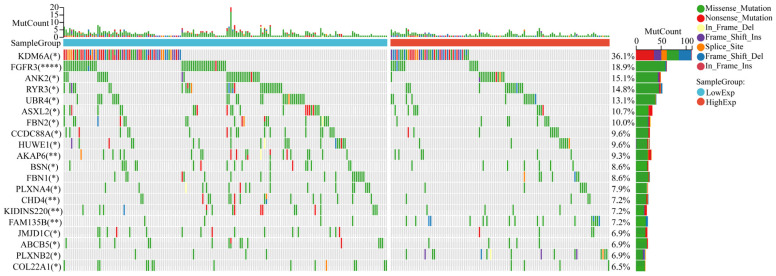
** The mutation profile in CCDC8 high and low groups.** Comparison of the mutational landscape between CCDC8 high group and CCDC8 low group. *P < 0.05 and **P < 0.01 and ****P < 0.0001.

The mutational landscape of bladder cancer samples was analyzed to compare the genetic alterations between the CCDC8 high group and the CCDC8 low group. The overall mutational count (MutCount) for each sample is displayed in the top bar plot, with different colors representing various mutation types, including missense mutations (green), nonsense mutations (red), frame shift insertions (orange), frame shift deletions (blue), splice site mutations (pink), in-frame insertions (purple), and in-frame deletions (cyan) (Figure 10).

The mutational profile suggests that in the CCDC8 high expression group, there is a notably lower frequency of mutations in genes such as FGFR3 and KDM6A.Instead, the CCDC8 high group shows a variety of mutations at different loci and of different types in other genes such as ANK2, RYR3, and UBR4. These mutations could influence tumor behavior through various mechanisms, including cell cycle regulation, signal transduction, and DNA repair pathways, contributing collectively to the aggressive phenotype seen in this subgroup.

## Discussion

The identification of CCDC8 as a prognostic marker in bladder cancer is consistent with its known role in other cancers. CCDC8 has been previously implicated in tumor progression, metastasis and chemotherapy resistance in several tumors^7^-^10^. Our findings extend these observations to bladder cancer, showing that high CCDC8 expression is linked to poor prognosis and a reduced response to immunotherapy.

The significant link between CCDC8 and the immune microenvironment in bladder cancer highlights the importance of understanding immune modulation in cancer therapy. Previous studies have shown that the tumor microenvironment is crucial in cancer progression and treatment response^14^-^16^. Our data indicate that CCDC8 may contribute to an immunosuppressive microenvironment, as evidenced by the upregulation of immune checkpoint molecules like PD-1, PD-L1, and CTLA-4, which are known to inhibit T cell function and facilitate tumor immune escape^17^-^19^. Furthermore, our findings suggest that targeting CCDC8 could improve the effectiveness of current treatments. Modulating the immune microenvironment and reducing immune suppression, CCDC8 inhibitors may improve responses to immunotherapy and other treatments. This is in line with previous studies showing that targeting components of the tumor microenvironment can enhance therapeutic outcomes 20, 21.

Furthermore, the functional enrichment analysis revealed that CCDC8 regulates pathways involved in metabolism, which is critical for tumor growth and survival. This is consistent with the literature indicating that metabolic reprogramming is essential for bladder cancer development and progression^22^-^24^.Metabolic reprogramming is a hallmark of cancer, allowing tumor cells to sustain rapid proliferation and survive under adverse conditions^25^.This metabolic shift supports the biosynthetic needs of rapidly dividing cells and contributes to an acidic tumor microenvironment, which can promote invasion and metastasis^26^, ^27^.The involvement of CCDC8 in metabolic reprogramming highlights its potential as a therapeutic target.

CCDC8 expression levels in bladder cancer are significantly associated with the mutation frequencies of various genes, notably FGFR3 and KDM6A. FGFR3 mutations are prevalent in low-grade, non-muscle invasive bladder cancers (NMIBC), but are less frequent in muscle-invasive bladder cancers (MIBC), which are prone to progression and metastasis^28^, ^29^.recent studies indicates that increased serine synthesis in FGFR3-mutant bladder cancer cells drives macrophages towards an immune-inert phenotype^30^, ^31^. Moreover, resistance to FGFR inhibitors in urothelial cancer involves mutations in the FGFR tyrosine kinase domain and modifications in the PI3K-mTOR pathway^32^. Combinations like erdafitinib-pictilisib or erdafitinib-gefitinib have shown potential to overcome this resistance^33^. In high CCDC8 expression bladder cancer, the lower frequency of FGFR3 mutations aligns with their more aggressive phenotype. This suggests these tumors rely on alternative oncogenic pathways. KDM6A functions as a tumor suppressor and is frequently mutated in high-grade, invasive bladder cancers^34^. These mutations disrupt gene regulation and chromatin remodeling, leading to uncontrolled cell growth^35^, ^36^. The lower frequency of KDM6A mutations in high CCDC8 tumors indicates reliance on different oncogenic mechanisms, diverging from the typical mutation pattern in high-grade tumors.

While our study identifies CCDC8 as a significant prognostic marker and promising therapeutic target in bladder cancer, further research is necessary to address our limitations and translate these findings into clinical practice. Future studies should focus on prospective validation, functional characterization, and therapeutic exploration to enhance our understanding and treatment of bladder cancer.

## Conclusion

Our study establishes CCDC8 as a key player in bladder cancer progression and immune regulation. These findings open new avenues for research and therapeutic strategies aimed at improving patient outcomes. Further investigation into the molecular mechanisms of CCDC8 and its interactions with other signaling pathways will be critical for developing targeted therapies.

## Supplementary Material

Supplementary figures and tables.

## Abbreviations

CCDC8: pro-apoptotic coiled-coil domain containing 8
BLCA: Bladder cancer
CUL7: Cullin 7
OBSL1: Obscurin-like 1
TCGA: The Cancer Genome Atlas
IC50: half maximal inhibitory concentration
GO: Gene Ontology
IHC: Immunohistochemical
KEGG: Kyoto Encyclopedia of Genes and Genomes
OS: overall survival
RFS: recurrence-free survival
BP: Biological Process
CC: Cellular Component
MF: Molecular Function
KRAS: Kirsten Rat Sarcoma Viral Oncogene Homolog
GZMA: Granzyme A
GZMB: Granzyme B
GZMM: Granzyme M
GZMH: Granzyme H
PRF1: Perforin 1
IFNG: Interferon Gamma
MDSC: Myeloid-Derived Suppressor Cells
BTLA: B and T Lymphocyte Attenuator
CTLA4: Cytotoxic T-Lymphocyte-Associated Protein 4
LAG3: Lymphocyte Activation Gene 3
PDCD1: Programmed Cell Death Protein 1
IDO1: Indoleamine 2,3-Dioxygenase 1
TGFB1: Transforming Growth Factor Beta 1
ARG1: Arginase 1
EDNRB: Endothelin Receptor Type B
HAVCR2: Hepatitis A Virus Cellular Receptor 2
VEGFA: Vascular Endothelial Growth Factor A
MHC: Major Histocompatibility Complex
SD/PD: Stable Disease/Progressive Disease
KDM6A: Lysine K Demethylase 6A
ANK2: Ankyrin 2
RYR3: Ryanodine Receptor 3
UBR4: Ubiquitin Protein Ligase E3 Component N-Recognin 4
