# Supplementary material for: Prognostic Significance of CCDC8 in Bladder Cancer: Insights from Bioinformatics and Immunohistochemical Analysis
Source: J Cancer. 2025 Jan 1;16(2):382–97. doi: 10.7150/jca.102655 (PMC11685694; doi:10.7150/jca.102655)
Supplement: Supplementary file 1 — Supplementary figures and tables. [file jcav16p0382s1.pdf]

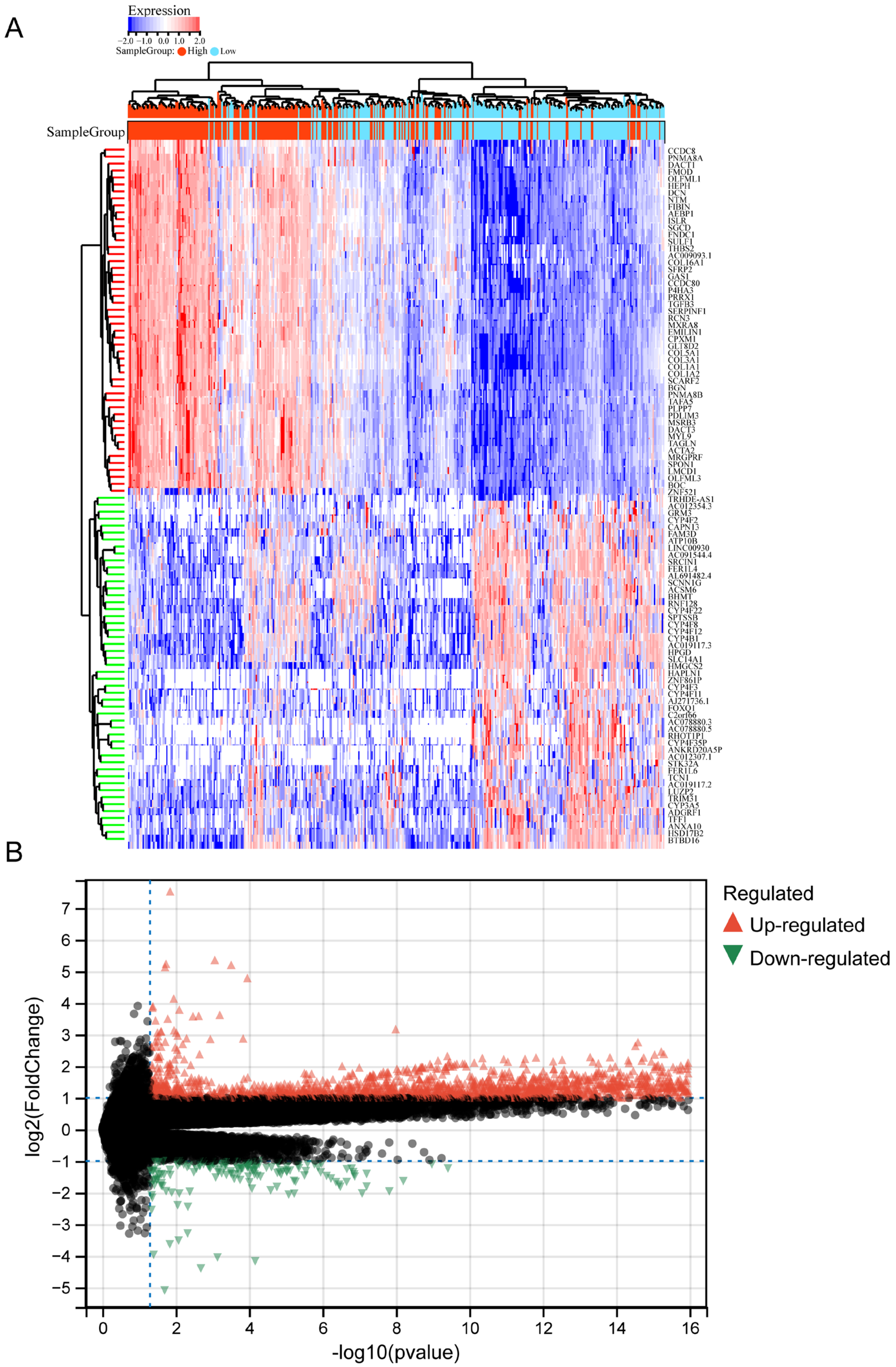

Figure S1. Screening of Differentially Expressed Genes (DEGs)

(A) Heatmap showing the expression levels of DEGs and(B) Volcano plot illustrating the DEGs.

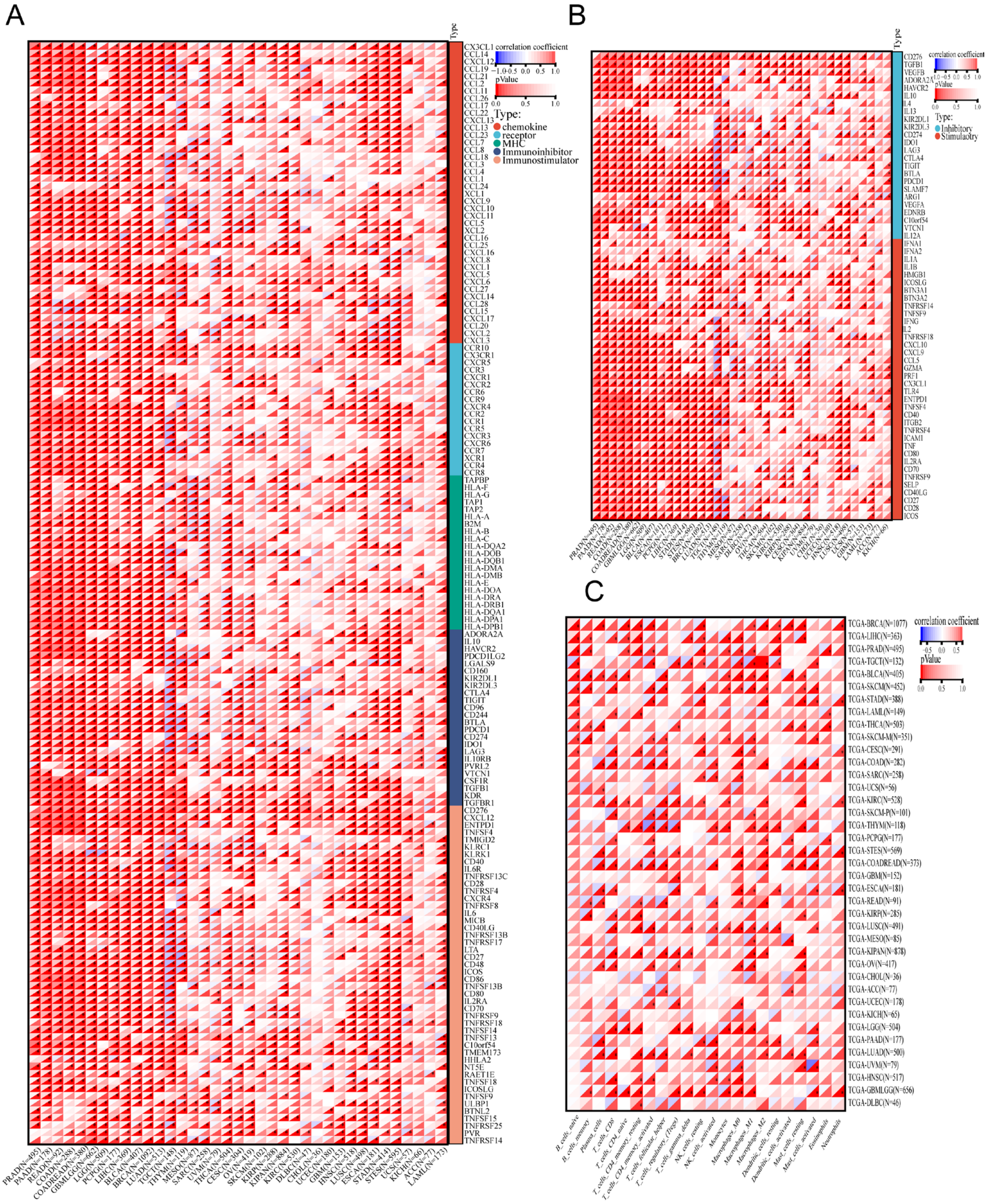

Figure S2. Immune regulation genes, immune checkpoint genes, and immune infiltration in pan-cancer cells are correlated with CCDC8 expression

CCDC8 expression is correlated with (A)five different immune regulatory genes, (B)two different immune checkpoint genes, and (C)22 types of immune cell infiltration. *P < 0.05

Table S1

TABLE S1 | Clinical information of patients in cohort 1. Bold indicates p-values less than 0.05.

Characteristics	Low(N=204)	High(N=204)	Total(N=408)	P-value
Age				**0.03**
<60	53(12.99%)	34(8.33%)	87(21.32%)	
>=60	151(37.01%)	170(41.67%)	321(78.68%)	
Sex				**0.04**
Female	44(10.78%)	63(15.44%)	107(26.23%)	
Male	160(39.22%)	141(34.56%)	301(73.77%)	
Grade				**0.02**
High	186(45.93%)	198(48.89%)	384(94.81%)	
Low	16(3.95%)	5(1.23%)	21(5.19%)	
Missing			3	
Stage				**3.6e-3**
I	2(0.49%)	0(0.0e+0%)	2(0.49%)	
II	80(19.70%)	50(12.32%)	130(32.02%)	
III	64(15.76%)	76(18.72%)	140(34.48%)	
IV	56(13.79%)	78(19.21%)	134(33.00%)	
Missing			2	

Table S2

TABLE S2 | Clinical information of patients in cohort 2. Bold indicates p-values less than 0.05.

Characteristics	Low(N=50)	High(N=50)	Total(N=100)	p-value
Age				0.67
<60	19(19.00%)	16(16.00%)	35(35.00%)	
>=60	31(31.00%)	34(34.00%)	65(65.00%)	
Sex				0.55
Female	5(5.00%)	8(8.00%)	13(13.00%)	
Male	45(45.00%)	42(42.00%)	87(87.00%)	
Stage				0.55
I/II	45(45.00%)	42(42.00%)	87(87.00%)	
III/IV	8(8.00%)	5(5.00%)	13(13.00%)	

Table S3

TABLE S3 | Clinical information of patients in cohort 3. Bold indicates p-values less than 0.05.

Characteristics	Low(N=174)	High(N=174)	Total(N=348)	p-value
Sex				0.24
Female	33(9.48%)	43(12.36%)	76(21.84%)	
Male	141(40.52%)	131(37.64%)	272(78.16%)	
Binary Response				**0.01**
CR/PR	44(14.77%)	24(8.05%)	68(22.82%)	
SD/PD	107(35.91%)	123(41.28%)	230(77.18%)	
Missing			50	
Enrollment IC				0.85
IC0	48(13.79%)	51(14.66%)	99(28.45%)	
IC1	65(18.68%)	67(19.25%)	132(37.93%)	
IC2	61(17.53%)	56(16.09%)	117(33.62%)	
Immune phenotype				0.24
Desert	45(15.85%)	31(10.92%)	76(26.76%)	
Excluded	63(22.18%)	71(25.00%)	134(47.18%)	
Inflamed	38(13.38%)	36(12.68%)	74(26.06%)	
Missing			64	

Table S4. Analysis of six different algorithms and bladder cancer signatures to correlate CCDC8 and molecular subtypes in cohort 1. Bold indicates p-values less than 0.05.

Characteristics	High(N=204)	Low(N=204)	Total(N=408)	p-value
Baylor subtype				**0.02**
Basal	83(20.34%)	60(14.71%)	143(35.05%)	
Differentiated	121(29.66%)	144(35.29%)	265(64.95%)	
UNC subtype				**1.3e-8**
Basal	119(29.17%)	61(14.95%)	180(44.12%)	
Luminal	85(20.83%)	143(35.05%)	228(55.88%)	
CIT subtype				**5.6e-20**
MC1	42(10.29%)	120(29.41%)	162(39.71%)	
MC2	22(5.39%)	3(0.74%)	25(6.13%)	
MC3	4(0.98%)	14(3.43%)	18(4.41%)	
MC4	39(9.56%)	0(0.0e+0%)	39(9.56%)	
MC5	1(0.25%)	0(0.0e+0%)	1(0.25%)	
MC6	3(0.74%)	1(0.25%)	4(0.98%)	
MC7	93(22.79%)	66(16.18%)	159(38.97%)	
Lund subtype				**7.6e-20**
Ba/Sq	30(7.35%)	23(5.64%)	53(12.99%)	
Ba/Sq-Inf	35(8.58%)	9(2.21%)	44(10.78%)	
GU	2(0.49%)	30(7.35%)	32(7.84%)	
GU-Inf	22(5.39%)	7(1.72%)	29(7.11%)	
Mes-like	26(6.37%)	8(1.96%)	34(8.33%)	
Sc/NE-like	12(2.94%)	5(1.23%)	17(4.17%)	
Uro-Inf	21(5.15%)	2(0.49%)	23(5.64%)	
UroA-Prog	22(5.39%)	72(17.65%)	94(23.04%)	
UroB	5(1.23%)	19(4.66%)	24(5.88%)	
UroC	29(7.11%)	29(7.11%)	58(14.22%)	
MDA subtype				**1.9e-15**
Basal	84(20.59%)	59(14.46%)	143(35.05%)	
Luminal	34(8.33%)	111(27.21%)	145(35.54%)	
p53-like	86(21.08%)	34(8.33%)	120(29.41%)	
TCGA subtype				**9.7e-21**
Basal squamous	82(20.10%)	51(12.50%)	133(32.60%)	
Luminal	25(6.13%)	21(5.15%)	46(11.27%)	
Luminal infiltrated	57(13.97%)	10(2.45%)	67(16.42%)	
Luminal papillary	28(6.86%)	118(28.92%)	146(35.78%)	
Neuronal	12(2.94%)	4(0.98%)	16(3.92%)	

Table S5. Analysis of six different algorithms and bladder cancer signatures to correlate CCDC8 and molecular subtypes in cohort 3. Bold indicates p-values less than 0.05.

Characteristics	High(N=174)	Low(N=174)	Total(N=348)	p-value
Baylor subtype				**4.4e-8**
Basal	130(37.36%)	79(22.70%)	209(60.06%)	
Differentiated	44(12.64%)	95(27.30%)	139(39.94%)	
UNC subtype				**7.7e-10**
Basal	106(30.46%)	48(13.79%)	154(44.25%)	
Luminal	68(19.54%)	126(36.21%)	194(55.75%)	
CIT subtype				**1.2e-18**
MC1	17(4.89%)	82(23.56%)	99(28.45%)	
MC2	3(0.86%)	7(2.01%)	10(2.87%)	
MC3	5(1.44%)	12(3.45%)	17(4.89%)	
MC4	85(24.43%)	16(4.60%)	101(29.02%)	
MC5	6(1.72%)	4(1.15%)	10(2.87%)	
MC6	2(0.57%)	0(0.0e+0%)	2(0.57%)	
MC7	56(16.09%)	53(15.23%)	109(31.32%)	
Lund subtype				**1.4e-18**
Ba/Sq	23(6.61%)	16(4.60%)	39(11.21%)	
Ba/Sq-Inf	26(7.47%)	4(1.15%)	30(8.62%)	
GU	3(0.86%)	24(6.90%)	27(7.76%)	
GU-Inf	14(4.02%)	17(4.89%)	31(8.91%)	
Mes-like	39(11.21%)	10(2.87%)	49(14.08%)	
Sc/NE-like	5(1.44%)	6(1.72%)	11(3.16%)	
Uro-Inf	28(8.05%)	2(0.57%)	30(8.62%)	
UroA-Prog	12(3.45%)	54(15.52%)	66(18.97%)	
UroB	9(2.59%)	18(5.17%)	27(7.76%)	
UroC	15(4.31%)	23(6.61%)	38(10.92%)	
MDA subtype				**1.8e-18**
Basal	64(18.39%)	37(10.63%)	101(29.02%)	
Luminal	22(6.32%)	102(29.31%)	124(35.63%)	
p53-like	88(25.29%)	35(10.06%)	123(35.34%)	
TCGA subtype				**3.4e-17**
Basal squamous	61(17.53%)	41(11.78%)	102(29.31%)	
Luminal	3(0.86%)	24(6.90%)	27(7.76%)	
Luminal infiltrated	97(27.87%)	46(13.22%)	143(41.09%)	
Luminal papillary	7(2.01%)	61(17.53%)	68(19.54%)	
Neuronal	6(1.72%)	2(0.57%)	8(2.30%)	
